# Our Journal

**Published:** 1888-12

**Authors:** 


### EDITORIALS AND MISCELLANEOUS.

OUR JOURNAL.—The present number closes the 18th volume of this journal. Since 1870 the editors have diligently labored to promote the medical literaruae of our section; to advance the progress of medical science, to maintain the honor of the profession and to aid and benefit the busy practitioner. That we have succeeded in a good degree, at least in all these points, we have abundant testimony in the continued success of the RECORD, and in the kind and encouraging words that mark the correspondence that comes up from all parts of the country. The 19th volume will commence with the January number 1889, and will in no respect be permitted to fall below its former efficiency and excellence. Indeed it will be the constant aim of the editors to increase the interest and practical usefulness of the RECORD in all departments, and to make it more and more the favorite of the busy practitioner. Let our friends write for us, talk for us and aid us in extending our circulation, and by so doing, enable us the better to improve the RECORD, thus benefiting themselves while helping us in advancing the medical progress and literature of our section.

